# Boosting Sensitivity of Cellulose Pressure Sensor via Hierarchically Porous Structure

**DOI:** 10.1007/s40820-025-01718-z

**Published:** 2025-03-31

**Authors:** Minzhang Chen, Xiaoni An, Fengyan Zhao, Pan Chen, Junfeng Wang, Miaoqian Zhang, Ang Lu

**Affiliations:** 1 College of Chemistry and Molecular Sciences, Wuhan University, Wuhan, 430072 People’s Republic of China; 2 School of Material Science and Engineering, Beijing Institute of Technology, Beijing, 100081 People’s Republic of China

**Keywords:** Pressure sensor, Cellulose, Hydrogel, High sensitivity

## Abstract

Pressure sensors are essential for a wide range of applications, including health monitoring, industrial diagnostics, etc. However, achieving both high sensitivity and mechanical ability to withstand high pressure in a single material remains a significant challenge. This study introduces a high-performance cellulose hydrogel inspired by the biomimetic layered porous structure of human skin. The hydrogel features a novel design composed of a soft layer with large macropores and a hard layer with small micropores, each of which contribute uniquely to its pressure-sensing capabilities. The macropores in the soft part facilitate significant deformation and charge accumulation, providing exceptional sensitivity to low pressures. In contrast, the microporous structure in the hard part enhances pressure range, ensuring support under high pressures and preventing structural failure. The performance of hydrogel is further optimized through ion introduction, which improves its conductivity, and as well the sensitivity. The sensor demonstrated a high sensitivity of 1622 kPa^−1^, a detection range up to 160 kPa, excellent conductivity of 4.01 S m^−1^, rapid response time of 33 ms, and a low detection limit of 1.6 Pa, outperforming most existing cellulose-based sensors. This innovative hierarchically porous architecture not only enhances the pressure-sensing performance but also offers a simple and effective approach for utilizing natural polymers in sensing technologies. The cellulose hydrogel demonstrates significant potential in both health monitoring and industrial applications, providing a sensitive, durable, and versatile solution for pressure sensing.

**Supplementary Information:**

The online version contains supplementary material available at 10.1007/s40820-025-01718-z.

## Highlights

- The study introduces a high-performance cellulose hydrogel (HPCH) with a biomimetic layered porous structure inspired by human skin, combining a soft layer with large macropores and a hard layer with small micropores. This unique design enhances both sensitivity and mechanical performance in pressure-sensing applications.
- The HPCH sensor demonstrates exceptional pressure sensitivity of 1622 kPa⁻^1^, a wide detection range of up to 160 kPa, and excellent conductivity of 4.01 S m^−1^. Ion immersion further optimizes the hydrogel’s conductivity and dielectric properties, offering superior performance compared to existing cellulose-based sensors.
- The sensor’s outstanding performance in health monitoring, industrial diagnostics, and pressure distribution detection positions it as a versatile, durable, and highly sensitive solution for various pressure-sensing applications, providing a promising path for natural polymer-based sensing technologies.

**Supplementary Information:**

The online version contains supplementary material available at 10.1007/s40820-025-01718-z.

## Graphical abstract

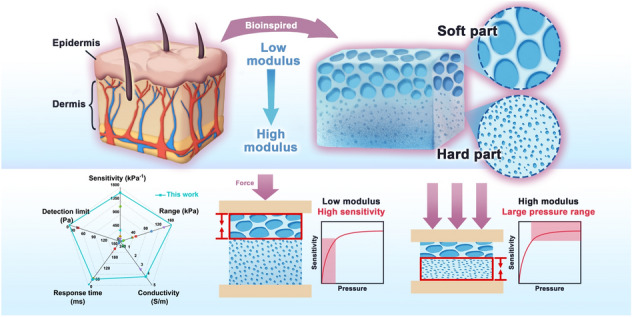

## Introduction

In recent years, due to the progress in technology and the growing demand for human health, flexible wearable devices have become increasingly prominent, showing a wide range of applications, such as human–machine interfaces, health monitoring fields and so forth [1–3]. As a kind of flexible wearable device, the pressure sensor can be divided into piezoelectric [4, 5], capacitive [6–8], piezoresistive [9–12], triboelectric [13] and optical [14] types according to the transduction mechanism. Different conduction mechanisms have their own advantages and disadvantages, and in capacitive pressure sensors the more variables affecting capacitance make them more sensitive to pressure changes. Also, they exhibit good repeatability, temperature independence, low power consumption [15, 16], and simple device structure, which has led to broader research. To further enhance the performance of capacitive pressure sensors, current research primarily focuses on optimizing the material or structure of dielectric layers and electrodes to improve the sensitivity and other important properties [17, 18]. For example, Ha et al. [17] devised a flexible hybrid-response pressure sensor composed of an electrically conductive porous nanocomposite (PNC) laminated with an ultrathin dielectric layer. Using a nickel foam template, the PNC was fabricated with carbon nanotubes doped Ecoflex to be 86% porous and electrically conductive, resulting in significantly enhanced sensitivity over wide pressure ranges, from 3.13 kPa^−1^ within 0–1 kPa to 0.43 kPa^−1^ within 30–50 kPa. Gao et al. [19] developed a Ti_3_C_2_T_*x*_-derived iontronic pressure sensor (TIPS) by taking the advantages of the high intercalation pseudo-capacitance under high pressure and rationally designed structural configuration. TIPS achieved an ultrahigh sensitivity (over 45,000 kPa^−1^) in a broad sensing range of over 1.4 MPa and low limit of detection of 20 Pa as well as stable long-term working durability for 10,000 cycles. Liu et al. [20] achieved superhigh maximum sensitivity of 9280 kPa^−1^ by using polyurethane-ionic liquid foam with a high porosity (95.4%) and a low modulus (3.4 kPa). Wang et al. [21] proposed a simple and rapid pre-treatment that allows the customization of the thickness of the polyvinylpyrrolidone (PVP) layer adsorbed on silver nanowires (AgNWs), improving the contact between conductors. The AgNWs were used in capacitive pressure sensors, demonstrating high transparency, sensitivity, and reproducibility.

However, most of the materials studied above are non-degradable or the degradation products are harmful to the environment, which are poorly biocompatible and lacking renewability. In contrast, cellulose, being the most abundant bioresource on Earth, is non-toxic, degradable, and environmentally friendly [22], capable of solving the above problems. It holds extensive applications and tremendous potential in flexible electronic devices [23]. Xiao et al. [24] prepared poly(3,4-ethylenedioxythiophene) dispersions using sulfonated nanocellulose, and constructed biocompatible sensors with a high sensitivity of 23.35 kPa^−1^, suitable for monitoring various mechanical strains. Hänninen et al. [25] mixed nanocellulose with microcrystalline chitosan to produce a greener piezoelectric film with a piezoelectric response of 2–8 pC N^−1^, offering a solution for the fabrication of environmentally friendly, low-cost piezoelectric films. Except sensory applications, cellulose also plays a significant role in constructing portable electrochemical energy storage devices [23], such as supercapacitors [26–29], lithium-ion batteries [30, 31], lithium-sulfur batteries [32, 33] and others. However, there are numerous hydrogen bonds and van der Waals forces in cellulose, which results in excessive modulus [23, 34, 35] and leading to reduced sensitivity ultimately when using cellulose as a raw material to prepare pressure sensor. However, high sensitivity sensors are needed in many fields. For example, real-time monitoring of human physiological signals (e.g., heart rate, blood pressure, etc.) in the field of health testing, detection of small pressure changes in the field of intelligent manufacturing for precision manufacturing and monitoring of the operating status of equipment. In response to these problems, the need to develop a cellulose pressure sensor with high sensitivity is imminent.

It is worth mentioning that human skins have gradient structures like the epidermal and dermal layers, which endow them with the ability to sense a wide range of pressures. By constructing bio-inspired gradient structures, not only the sensitivity of flexible pressure sensors can be improved, but also their detection range can be further expanded [36]. Therefore, inspired by the gradient structure of skin, a cellulose pressure sensor with similar structure to human skin was proposed, which has a hierarchically porous structure composed of a low-modulus soft layer and a high-modulus hard layer, to achieve boosted sensitivity and expanded pressure detection range. In this work, bio-inspired hierarchically porous cellulose hydrogels (HPCH) have been developed. In the unique hierarchical structure, the loosely porous layer with low modulus enabled the cellulose sensor to show a dramatic change of capacitance when subjected to low pressure, which effectively increased the sensitivity and lowers the limit of detection. On the other hand, the densely microporous layer improved the modulus and compressive strength of the cellulose hydrogel, ensuring a stronger compression resistance and realizing an overall high sensitivity and wide pressure range. As a result, the cellulose hierarchical hydrogel can be successfully applied to pressure sensor, with sensitivity up to 1622 kPa^−1^, minimum detection limit of 1.6 Pa, response time of 33 ms, detection range up to 160 kPa, as well as excellent repeatability and durability, surpassing most reported cellulose-based sensors. As a consequence, the present study not only supplied an effective bio-inspired strategy to accomplish a qualitative leap in sensitivity of pressure sensors, but also expanded the potential application of cellulose hydrogels in a variety of fields including medical therapy, human–machine interface, etc.

## Experimental Section

### Materials

Cotton linter pulp as cellulose source was supplied by Hubei Golden Ring Co., Ltd. (Xiangfan, China). Its viscosity-average molecular weight was measured to be 10.8 × 10^4^ g mol^−1^, as previously reported [37]. The cellulose was vacuum dried at 60 °C for 48 h to remove any moisture. Benzyltrimethyl ammonium hydroxide (BzMe_3_NOH, 40 wt% aqueous solution) was purchased from Sigma-Aldrich (Shanghai, China) Trading Co., Ltd. Other reagents including epoxy chloropropane, tween 80, isooctane, KCl, LiCl and trisodium citrate (C_6_H_5_Na_3_O_7_) of analytical grade were purchased from Sinopharm Chemical Reagent Co., Ltd., and used without further purification.

### Preparation of the HPCH

The cellulose sample was dispersed in 1.88 mol L^−1^ BzMe_3_NOH aqueous solution and the concentration of cellulose was kept as 3.0 wt%. After frozen overnight at −24.0 °C for 12 h, the mixture was thawed at room temperature, to obtain a transparent solution. The cellulose solution (50 g) was placed in cold trap and 2 mL epoxy chloropropane was added with mechanical stirring. After stirring with 1000 rpm for 1.5 h, 4 g Tween 80 and 15 mL isooctane was added, followed by stirring at 900 rpm for another 1 h. Then the obtained solution was divided into two portions: one was centrifuged with 5000 rpm for 5 min to remove air bubbles, then poured into a mold to form the hard layer. The other portion was sonicated by ultrasonic cleaner type KQ2200E at 40 kHz for 3 min and poured onto the hard layer to form the soft layer. After standing for 1.5 h, a hydrogel with a layered, porous structure can be obtained. The hydrogel was soaking in 50.0 v% ethanol for 2 days to remove the excess residual solvent. Finally, the hydrogel was immersed in 1.0 mol L^−1^ electrolyte solutions for 2 days, such as potassium chloride (KCl), lithium chloride (LiCl) and trisodium citrate, to enabling ionic conductivity.

### Characterization

The mechanical property of HPCHs were measured on a universal tensile-compressive tester (CMT 6503, Shenzhen SANS Test Machine, Shenzhen, China). For the compression test, hydrogel samples were cut into a square shape with sides of 1.0 cm and tested at a compression rate of 2.0 mm min^−1^ at 25.0 °C and 60% relative humidity. All samples were subject to at least 5 parallel tests. The Young’s modulus was calculated from the initial linear region of the stress–strain curves. Fourier transform infrared (FT-IR) spectra of HPCHs were acquired on an FT-IR spectrometer (5700 FT-IR Spectrometer, Thermo Fisher Scientific) over a wavelength range from 4000 to 400 cm^−1^ with 2 cm^−1^ resolutions. The ionic conductivity of the cellulose hydrogel samples was measured by the electrochemical impedance spectra (EIS) with a voltage amplitude of 5 mV and a frequency range from 100 kHz down to 1 Hz on an electrotechnical workstation (CHI760E, CH Instruments Ins). The ionic conductivity (*σ*) was then calculated according to Eq. (1):

1 $$s = \, l/\left( {R \times S} \right)$$

where *l* is the sample thickness, *R* is the intercept of the abscissa in the Nyquist plot representing the internal resistance of the sample, and* S* is the area of the electrode. X-ray diffraction (XRD) pattern of the HPCH was recorded with a Rigaku Miniflex600 diffractometer in reflection mode with Cu Kα radiation (*λ* = 0.154 nm) and the scanning speed was 5 min^−1^. Scanning electron microscopy (SEM) images were taken with the field emission scanning electron microscopy (Zeiss, Sigma, England) using an accelerating voltage of 5 kV. A capacitance meter (Capacitance Tester, UC2652, UCE Technologies) was applied to record the capacitance change of the HPCH under pressure, with Ag or Cu foil as electrodes. The maximum voltage of the sinusoidal measurement signal was set as 1 V, and the frequency remained at 1000 Hz. For capacitive pressure sensors, sensitivity (*S*) can be expressed as:

2 $$S \, = \delta (DC/C_{0} ) \, /\delta P$$

where *C*_0_ is the initial capacitance before applying pressure (*P*), and *ΔC* = *C*−*C*_0_ presents the change of capacitance upon loading.

### Assembly of the HPCH-Based Sensor and Performance Characterization

The HPCH-based sensor was assembled for the monitoring of human physical behavior. The HPCH samples were cut into rectangular shapes and packaged with VHB (VHB 4905, 3 M) on both sides, with Ag or Cu foil as electrode. The thickness of the HPCH and VHB are about 1.5 and 0.5 mm, respectively. Afterward, the HPCH sensor was fixed on the human skin, such as the eyebrow, mouth, neck, wrist, or knees, to monitor the physical behavior of humans. Informed written consent was obtained from participants for all studies (including experiments using sensors or wearable technology).

To further demonstrate the potential application, the HPCH sensor was used for detecting the work status of machines. The HPCH samples were cut into rectangular shapes and packaged with VHB (VHB 4905, 3 M) on both sides, with Ag or Cu foil as electrode. The thickness of the HPCH and VHB were about 1.5 and 0.5 mm, respectively. Then, the HPCH sensor were pasted on the surface of a hair dryer or pump (such as water pump). The fault scenario was simulated and the capacitance variation of the HPCH was detected. Finally, the curve of capacitance change over time was transformed into a more intuitive waveform by wavelet transform.

To ensure the consistency of the sensor array, a 4 × 4 sensor array was prepared consisting of 16 individual HPCH-based pressure sensors. Each sensor cell in the array was fabricated using hydrogel of uniform thickness and precise dimensions (10 mm × 10 mm) and carefully cut using a mold to ensure consistency across all sensor cells. The array was assembled into a VHB encapsulation layer with copper foil electrodes on the top and bottom, further ensuring uniform electrical contact across the array. The insole shaped arrays were prepared in a similar process.

## Results and Discussion

The concept of biomimicry involves emulating natural structures to replicate their functional properties, providing a pathway to develop advanced materials with tailored characteristics. In nature, many biological tissues exhibit gradient structures that contribute to their unique mechanical properties and functionalities. For example, the human dermis displays a gradient in Young's modulus and stiffness from top to bottom, enabling a broad pressure sensing range from approximately 1 kPa to 1 MPa [15, 38]. Inspired by this natural design, we have developed a hierarchical porous cellulose hydrogel (HPCH) that mimics the dermal structure by incorporating both soft and hard layers, for the designation of boosted sensitive pressure sensor.

The preparation process of HPCH is shown in Fig. S1. A transparent cellulose solution was prepared, and epichlorohydrin was added as a cross-linking agent, followed by the addition of Tween 80 and isooctane with stirring to form an oil-in-water emulsion. The mixture was then divided into two parts, one part was centrifuged to remove air bubbles and poured into a mold to form a uniform, hard layer, and the other part was ultrasonicated for 3 min to eliminate larger bubbles, leaving only tiny bubbles of consistent pore size. This was then poured over the hard layer in the mold, creating a loose, soft layer. The entire structure was left undisturbed for 1.5 h, allowing the cellulose to cross-link with epichlorohydrin and form a hydrogel. The hydrogel was then immersed in ethanol to remove the oil phase and an ion solution to enable ionic conductivity, respectively, to fabricate hydrogel with hierarchically porous structure. Finally, the HPCH was used to prepare the pressure sensor.

### Designation and Mechanistic Explanation of HPCH

**Fig. 1:**
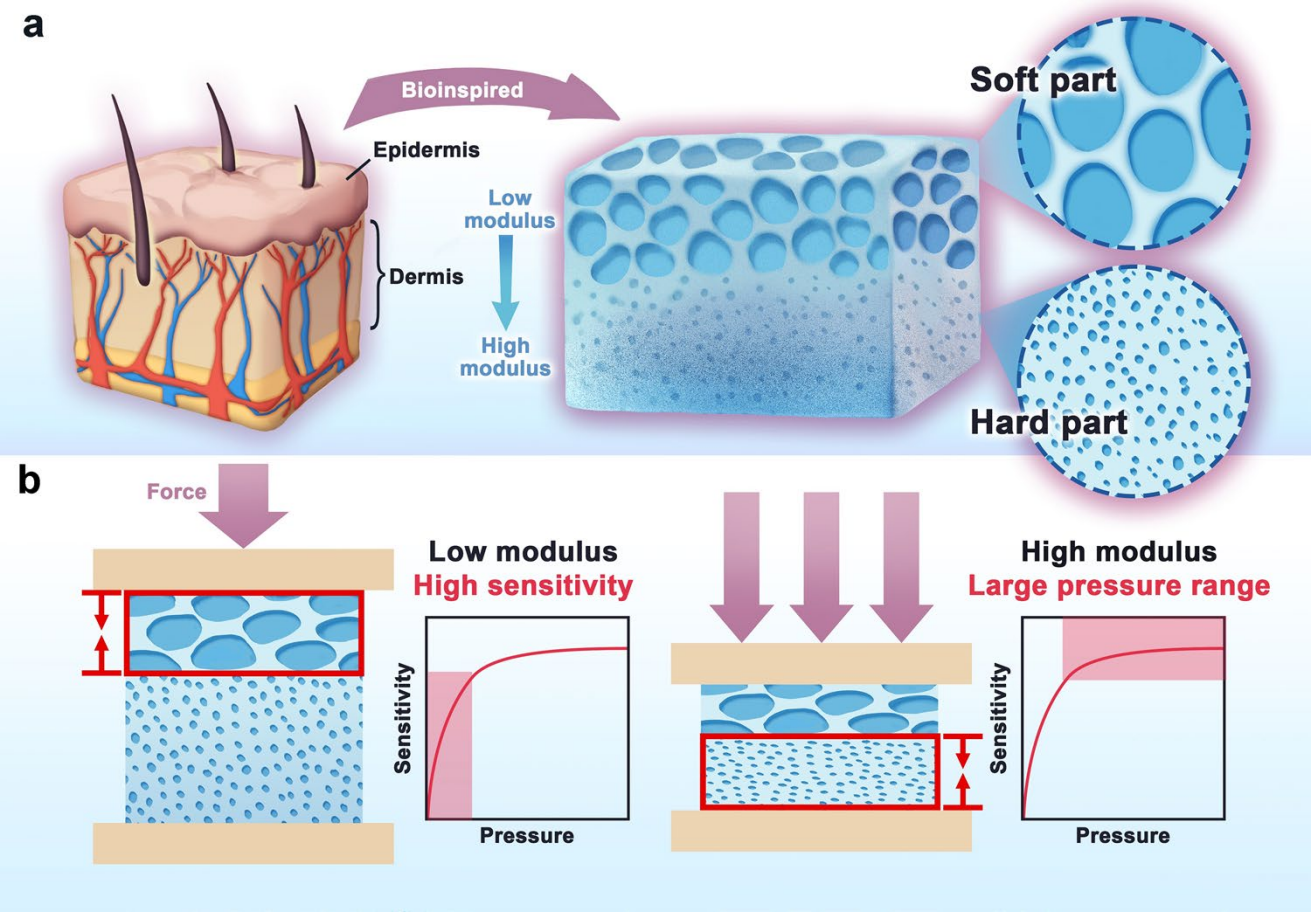
Designation of cellulose hydrogel for boosted sensitivity and large pressure range via hierarchically porous structure. **a** Microstructure of human skin and the structure of the bio-inspired gradient structure of HPCH. **b** Mechanistic explanation of HPCH when subjected to different pressure

As presented in Fig. 1a, the structure of this HPCH is inspired bionically and designed rationally to replicate the layered composition of the dermis. The upper layer of the hydrogel is not only soft, but also loosely porous, formed by the solution of product with tiny bubbles of similar pore size. This soft, flexible layer provides high sensitivity to low-pressure stimuli, allowing for the detection of gentle touches and minor deformations, much like the upper regions of the dermis, i.e., epidermis. In contrast, the underlying hard layer is dense and rigid, with micron-sized pores that are invisible to the naked eye. This layer is formed from a homogeneous solution, following centrifugation to remove air bubbles. In this process, the oil phase (e.g., Tween 80) was divided into small droplets that are uniformly dispersed throughout the hydrogel. When the hydrogel was immersed in ethanol, the oil-phase structures were washed away, leaving behind a microporous structure. This dense, hard layer enhances the overall structural integrity of the HPCH, enabling it to withstand and respond to larger pressures, similar to the deeper, stiffer regions of the dermis. The synergistic effect of these layers results in a hydrogel with a broad pressure-sensing range, capable of detecting and adapting to various mechanical stimuli, effectively mimicking the functional characteristics of human skin.

The HPCH with a different layered structure had a surprising phenomenon when subjected to external pressure. As shown in Fig. 1b, when the hydrogel is subjected to very weak pressure, it is the soft part of the upper layer first dominates, whose modulus is much lower. And when the pressure gradually become larger, the hard part in lower layer begin to take the leading role. Obviously, this strategy reduced the modulus of the hydrogel by changing the aggregation structure, which could increase the sensitivity of a capacitive sensor according to Eq. (3):

3 $$C = \frac{{\varepsilon_{0} \varepsilon_{{{\text{re}}}} S/d}}{1 - P/E}$$

where *C* is capacitance, *ε*_0_ is the vacuum permittivity, *ε*_re_ is the relative permittivity of cellulose hydrogel, *S* is the surface area of cellulose hydrogel, *d* is the thickness of cellulose hydrogel, *E* is the modulus of cellulose hydrogel. On the other hand, it is worth mentioning that the sensitivity can be further improved by a simple ion-soaking strategy, such as soaking the hydrogel in lithium chloride, potassium chloride, trisodium citrate, and other electrolyte solutions in order to increase the value of the relative dielectric constant and thus the sensitivity, as shown in Eq. (4)

4 $$C = \frac{{ \varepsilon_{{{\text{re}}}} S}}{4 \pi k d}$$

where *k* is the electrostatic constant. The sensitivity of HPCH was improved significantly through these methods, which will be described in the following part in detail.

### Characterization and Deformation Mechanisms of HPCH

**Fig. 2:**
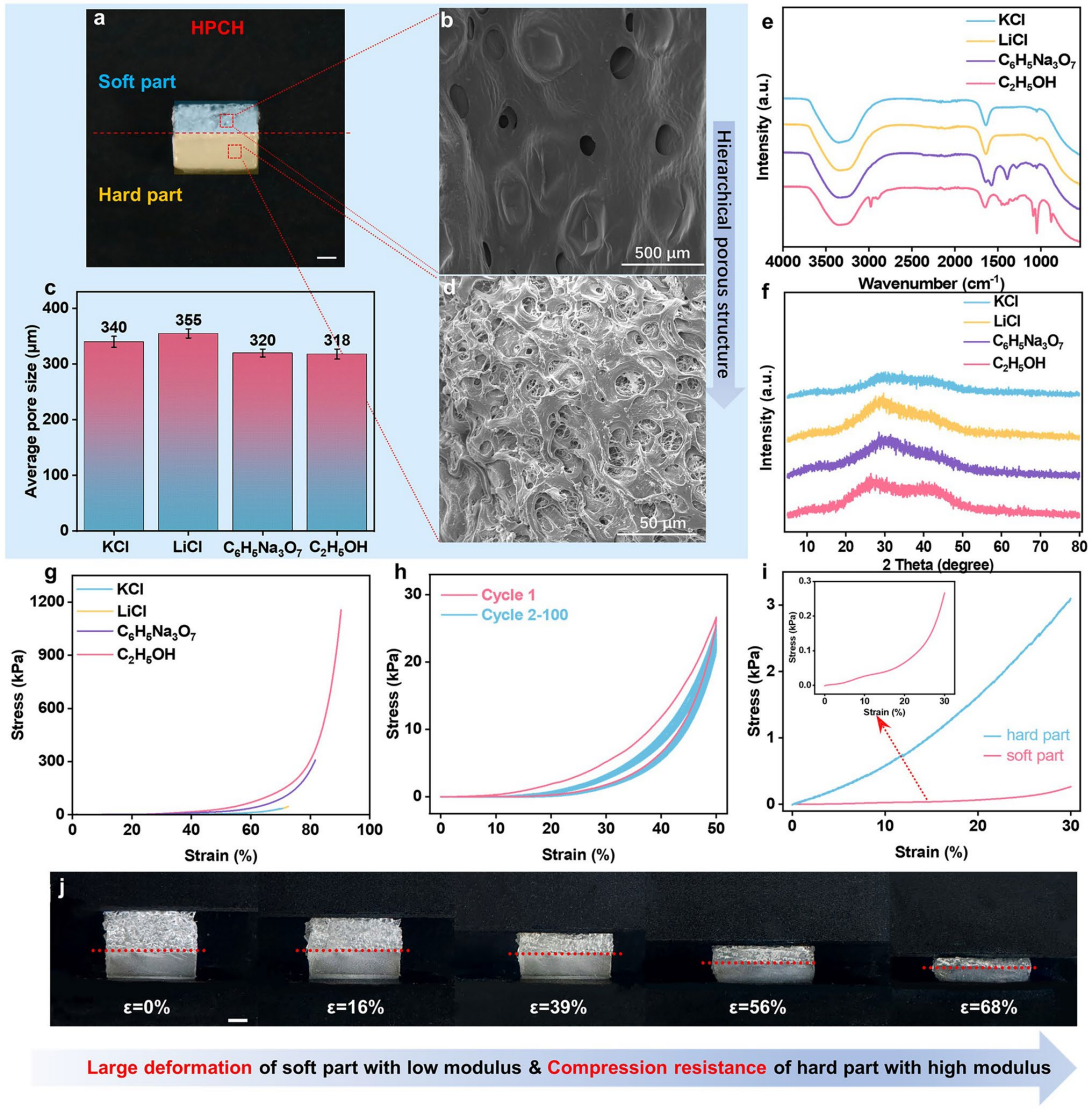
**a** Physical picture of HPCH (hard part is marked blue and soft part is marked yellow; scale bar: 1 mm). **b** SEM images of the soft part of HPCH. **c** Average pore size in the upper portion of HPCHs immersed in different solutions. **d** SEM images of the hard part of HPCH. **e** FT-IR spectra of the HPCHs immersed in different solutions. **f** XRD patterns of the HPCHs immersed in different solutions. **g** Compressive stress–strain curves of HPCHs immersed in different solutions. **h** Compressive stress–strain curves of HPCH immersed in C_6_H_5_Na_3_O_7_ solution within 50% strain during loading–unloading cycles. **i** Compressive stress–strain curves for soft and hard part of HPCH immersed in C_6_H_5_Na_3_O_7_ solution. **j** Optical physical image of HPCH during compression with different strains in the range of 0–68% (Scale bar: 1 mm)

As shown in Fig. 2a, the overall structure of the hydrogel can be clearly seen through the photograph, with the upper layer labeled blue as the soft part and the lower layer labeled yellow as the hard part. The HPCH's layered porous structure is then completely reflected by the macropores carried by the loose, soft part and the micropores in the dense, hard part, as seen in Fig. 2b, d. Meanwhile, optical microscopy diagrams of the upper portion of the hydrogel are shown in Fig. S2, where it could be observed that the macropores in the soft portion are all at the level of a few hundred micrometers without great variation. The pore size of these macropores in the soft part was calculated, and their average pore sizes are shown in Fig. 2c, which were in the range of several hundred of micrometers. The pore sizes of the hydrogels immersed in different electrolytes were larger than the one immersed in ethanol, which indicates a slight swelling and the enlargement of the pores in aqueous solution.

Figure 2e depicts the FT-IR spectra of HPCHs soaked in different solutions (ethanol, KCl, LiCl, and trisodium citrate). These cellulose hydrogels were found to have broad bands at 3300–3500 cm^−1^ (assigned to O–H stretching) and around 1050 cm^−1^ (assigned to C–O stretching). And the 1419 cm^−1^ band was attributed to –CH_2_ stretching of cellulose crystalline II structure. This band is widely recognized in the literature as indicative of the ordered molecular arrangement in cellulose crystals [39–41]. What’s more, after being submerged in the three ion solutions, the C–H absorption peak around 2900 cm^−1^ vanished because the interaction between with ion and cellulose altered the configuration of the intermolecular hydrogen bonds while also masking the strength of the C–H vibrations, making the C–H signal invisible. This indicated that ionic solutions exchange had only physical effects. More importantly, as shown in Fig. 2f, the XRD peak at 2*θ* = 28.5° originated from the regenerated cellulose crystalline planes (101) and (002), which indicated the cellulose II crystal structure. It is shifted with respect to the standard diffraction angle because the ionic and ethanol solutions may cause the crystalline planes in the cellulose II structure to align more closely, which would increase the interaction force between the cellulose molecular chains and lead to a slight contraction of the lattice spacing. According to Bragg's law 2*d* sin*θ* = *nλ*, when the lattice spacing *d* decreased, the diffraction angle 2*θ* increased, so the main peak was shifted to a higher angle. This occurrence lends credence to FT-IR's finding that solution treatments primarily affect the cellulose structure physically rather than chemically. Besides, Fig. S3 depicted the comparison of electrical conductivity of HPCHs. Evidently, immersing the hydrogel in ion solution would improve its conductivity significantly. And the hydrogel immersed in KCl displayed the highest electrical conductivity because of the fastest ion migration rate compared LiCl and trisodium citrate. This was mainly due to the fact that potassium ions have a relatively small hydration radius and are able to move faster inside the hydrogel, increasing the overall conductivity. It was worth mentioning that all the structural changes brought about by the solvent exchange to the hydrogel were physical and did not involve chemical reactions. During the immersion in electrolyte solution, ions entered the hydrogel through physical interactions and then the hydrogel swelled because of the difference in internal and external osmotic pressure, which also led to the difference in their mechanical properties. As shown in Fig. 2g, HPCH immersed in ethanol exhibited much higher compressive strength, due to that ethanol could remove water molecules from the hydrogel, leading to an increase in hydrogen bonding and van der Waals forces between the cellulose chains in the hydrogel [42], which made the structure of the hydrogel more compact and stiff. In addition, the tensile stress–strain curve of HPCHs is shown in Fig. S4, which could satisfy the basic tensile aspect. Figure 2h shows the compressive stress–strain curves of HPCH at 50% strain during loading–unloading cycles. In the first cycle, a certain hysteresis was observed for HPCH, and this hysteresis phenomenon was a reflection of partial energy dissipation. The hysteresis was likely due to the internal rupture of irreversible cross-linking between cellulose, along with the dissociation of chain entanglements and disruption of hydrogen bonding during the first loading and unloading cycle, similar to that previously reported for polysaccharide-based hydrogels [43]. Specifically, the mechanical property of the soft and hard parts of the HPCH soaked in C_6_H_5_Na_3_O_7_ solution were tested separately, as shown in Fig. 2i. It can be clearly seen that the soft part was subjected to much less stress than the hard part when compressed to the same strain. Therefore, the soft part would take the dominant role when HPCH is first subjected to tiny pressure. Afterward, when the pressure continued to increase beyond the pressure limit of the soft part, the hard part would take the leading role.

To investigate the deformation mechanism of the HPCH with soft and hard parts under compression, morphological observations were performed using a camera during compression at different strain levels (Fig. 2j). Initially, when the hydrogel was subjected to external pressure, the soft layer underwent the majority of the deformation, resulting in a significant increase in compression strain. For example, as shown in Table S1, when the overall strain of the hydrogel reached 16%, the soft layer was compressed by 21%, while the hard layer experienced only 4% deformation. As the strain increased to 39%, the deformation of the soft layer reached 50%, while the hard layer was compressed by 13%. At 56% overall strain, the soft layer’s deformation was 62%, with the hard layer deforming by 32%. Finally, when the strain reached 68%, the soft layer gradually saturated with a deformation of 83%, and the hard layer deformed by 55%. This mechanism is further supported by the SEM images in Fig. S5, which show the gradual compression of the soft layer's pores, while the hard layer retains its microstructure even at higher strains, demonstrating its ability to maintain its structural integrity under pressure.

To summarize, the deformation of HPCH gradually transferred from soft part to hard part with increasing compression with increasing the pressure, due to the different modulus. Importantly, this phenomenon of strain transfer and gradient compression under high strain compression conditions was expected to significantly enhance the wide range pressure sensing capability of hydrogel sensors.

### Sensitivity and Sensing Properties of HPCH

**Fig. 3:**
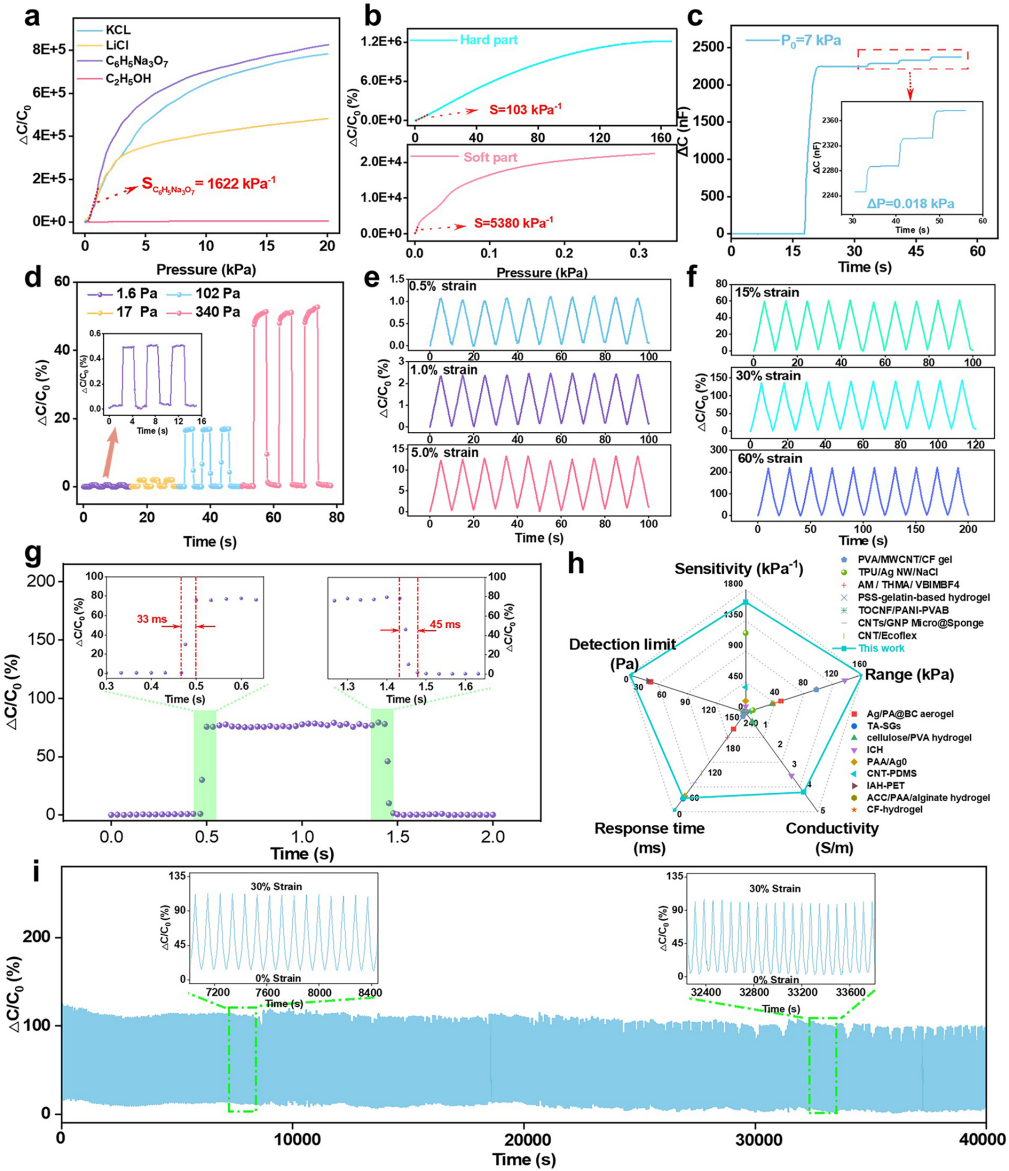
**a** Δ*C*/*C*_0_ variations of HPCHs immersed in different solutions. **b** Δ*C*/*C*_0_ variations and sensitivity (*S*) of soft and hard part of HPCH immersed in C_6_H_5_Na_3_O_7_ solution. **c** Change in capacitance with continued application of a small pressure in a large pressure situation. **d** Relative capacitance changes of HPCH sensor under different compressive stress. Relative capacitance changes of HPCH sensor under different compressive strains during 10 successive cycles: **e** small strain, and **f** large strain. **g** Response time and release time of HPCH sensor. **h** Sensitivity, detection limit, response time, conductivity and pressure range of HPCH compared to other materials [17, 37, 44–57]. **i** Relative capacitance changes under repeated loading–unloading processes with a strain of 30% for 5000 cycles, showing the durability of the sensor

Factors such as the decrease in the modulus of the hydrogel and the increase in conductivity mentioned above had a significant effect on its sensitivity. For comparison and better aesthetics, we calculated the sensitivity over the pressure range of 0–20 kPa, as shown in Fig. 3a (all complete curves were shown in Fig. S6). The sensitivity (0–2.9 kPa) of the hydrogel immersed in ethanol only is 9.8 kPa^−1^, while after ion introduction, the sensitivity is significantly increased to more than 1000 kPa^−1^, where *S*_trisodium citrate_ = 1622 kPa^−1^ within 0–2 kPa, *S*_KCl_ = 1169 kPa^−1^ within 0–0.8 kPa, and *S*_LiCl_ = 1257 kPa^−1^ within 0–1.2 kPa. The reason for this was that, in addition to the increase in the conductivity and decrease in the modulus of the hydrogel, the introduction of ions can also change the dielectric constant of the hydrogel and increase its ability to respond to electric fields. The interaction between ions and cellulose chains as well as the double layer effect formed on the surface and inside of the hydrogel can enable the hydrogel to store more charge as well as the presence of ions can enhance the polarization effect of the hydrogel. These mechanisms allow the capacitance value of the hydrogel to change more dramatically when subjected to pressure, thus increasing the sensitivity of the sensor. Additionally, all three groups of ion-soaked samples were able to withstand pressure exceeding 100 kPa, with the sodium citrate-soaked HPCH demonstrating the highest resilience, enduring pressure up to 160 kPa.

It is interesting to note that the hydrogel soaked in trisodium citrate solution had the highest sensitivity. We speculated that this might be due to the higher initial conductivity of the hydrogel soaked in KCl and LiCl solution. Therefore, the change of capacitance (△*C*/*C*_0_) was smaller in the compression process, which leads to the lower sensitivity according to Eq. (2). Subsequently, in order to gain deeper insights into the mechanisms behind the increased sensitivity, we focused on the hydrogel with the highest sensitivity which was soaked in trisodium citrate solution. As seen in Fig. 3b, we specifically analyzed the sensitivity of its soft and hard part: *S*_soft part_ = 5380 kPa⁻^1^ (within 0–0.01 kPa), *S*_hard part_ = 103 kPa⁻^1^ (within 0–10.6 kPa). When these two parts were combined in the HPCH, the overall sensitivity was measured as 1622 kPa⁻^1^. The difference in sensitivity between the soft and hard parts of the hydrogel could be attributed to their distinct structural and mechanical properties. The soft part, with its higher deformability and lower modulus, was more responsive to small pressure change, offering high accuracy in detecting subtle variations. The increased surface area due to its larger pore size also contributed to this heightened sensitivity by allowing more significant charge accumulation and polarization under pressure. In contrast, the hard part, with its higher modulus, was less deformable, resulting in lower sensitivity. However, the hard part provided crucial structural support, ensuring the hydrogel maintains its integrity and function under higher pressure, where the soft part might otherwise become overly compressed. When these two parts were combined, a synergistic effect balanced the overall sensitivity of the hydrogel and detection range. Each contributed differently, yet together they created a material with a broad pressure-sensing range that leveraged both the high sensitivity of the soft part and the structural ability to withstand high pressure of the hard part. This synergy resulted in a balanced and versatile sensing capability, making the hydrogel effective across a wide range of pressures and ideal for applications that demand both high sensitivity and robust pressure range.

In order to better validate the potential of HPCH in the field of sensing, a series of tests on sensing were conducted. As shown in Figs. 3c and S6, the capacitance change could be observed obviously when applying continuous micropressure (0.018 kPa) under a large pressure of 7 kPa. The result confirmed high sensitivity of the HPCH and the change of micropressure could be detected rapidly and evidently under a large pressure. Moreover, to investigate the lowest detection limit of HPCH, the pressure applied over the hydrogel was reduced continuously until the minimum pressure detected by the capacitance detector, as shown in Fig. 3d. The lowest detection limit was 1.6 Pa, which was also a reflection of high sensitivity. Then, in order to demonstrate that HPCH deformation had good repeatability of capacitance change, several groups of cyclic experiments of HPCH were carried out. From Fig. 3e, f, the cellulose sensor could detect the change of capacitance quickly and instantly regardless of whether large (60%) or small (0.5%) strain occurred and the capacitance signal changed stably with good repeatability. It was proved that the hydrogel had good performance of repeated compression and real-time response. In addition, at a low pressure of 1 kPa, corresponding to the pressure of a gentle finger touch, HPCH indicated a rapid response and relaxation time of 33 and 45 ms, respectively, as shown in Fig. 3g, which were comparable to the response time of human skin (30–50 ms). More importantly, we compared HPCH with other materials in terms of sensitivity, detection limit, response time, conductivity and pressure range, as shown in Fig. 3h. The comparative data of the properties of different materials in Fig. 3h are listed in Table S2. It could be clearly seen that the HPCH in this work has superior performance than its counterparts in all these aspects, which reflects the good application potential of HPCH. Besides, the HPCH sensor also presented rapid and excellent sensing response consistency during 5000 cycles of 30% strain (Fig. 3i), and the capacitance change ratio remained constant under repeated loadings, demonstrating its remarkable electrical performance reliability and durability when employed in practical applications. Moreover, our HPCH sensor is also insensitive to temperature and humidity, as shown in Fig. S8. In summary, our strategy for preparing hydrogels with high sensitivity and high sensing properties provided a simple and feasible idea that offered great potential for the application of natural polymers in the field of sensing.

### Application of HPCH in the Field of Health Monitoring and Mechanical Fault Diagnosis

**Fig. 4:**
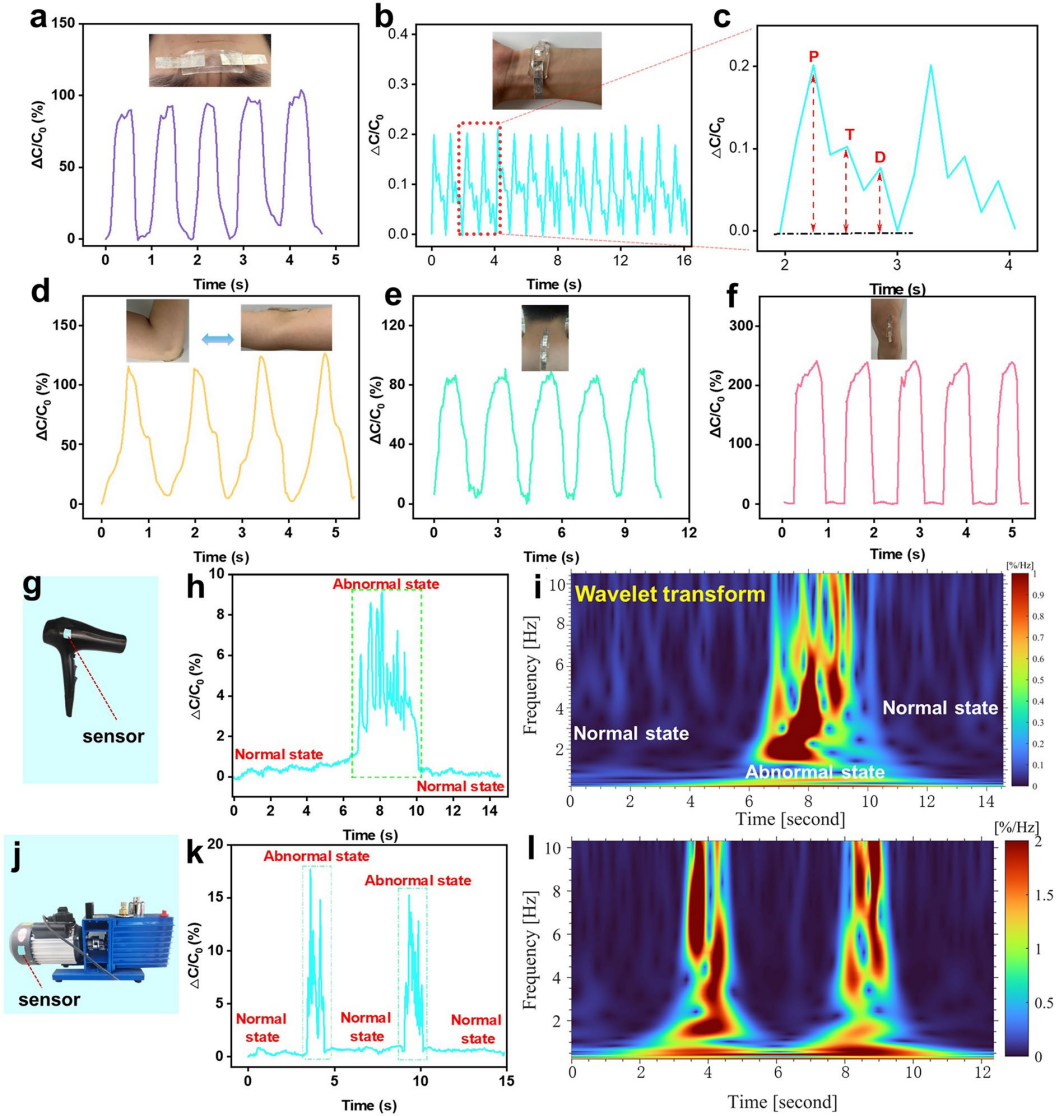
**a** Corresponding capacitive signals of frowning. **b** Corresponding capacitive signals of wrist pulse. **c** Corresponding capacitive signals of one pulse with percussive wave (*P*-wave), tidal wave (*T*-wave), and diastolic wave (*D*-wave). The corresponding capacitive signals of **d** elbow, **e** neck and **f** knee. **g** Photograph of the sensor attached onto the hairdryer. **h** Capacitive signals from hairdryer vibrations detected by the sensor. **i** Frequency-time window of capacitive signals from hairdryer vibrations obtained by using wavelet transform. **j** Photograph of the sensor attached onto the pump. **k** Capacitive signals from pump vibrations detected by the sensor. **l** Frequency-time window of capacitive signals from pump vibrations obtained by using wavelet transform

The above performance characterizations all proved the high sensitivity and fast responsiveness of HPCH, which were appropriate for using as a sensor to detect subtle movements, such as pulse, frown and body bending and so on. Thus, the HPCHs were cut into rectangular shape and packaged with VHB on both sides, with Ag foil as the electrode to prepare pressure sensor. The sensor was applied to the eyebrows of volunteers to monitor the change of eyebrows as shown in Fig. 4a, and the frowning gesture was successfully detected, which was beneficial for reading mental activities as well. Additionally, we tested the hydrogel's application in other area of the body, such as the wristband to monitor the pulse, as shown in Fig. 4b. The pulse is a vital physiological signal, providing insights into heart rate as well as systolic and diastolic blood pressure. With a frequency of 68 beats per minute, the HPCH sensor demonstrated its ability to consistently capture regular and repeatable pulse patterns, corresponding to the expected values for healthy adults at rest. Moreover, as illustrated in Fig. 4c, a close-up of a single pulse peak clearly revealed the characteristic features of the pulse waveform, including the percussive wave (*P*-wave), tidal wave (*T*-wave), and diastolic wave (*D*-wave). These well-defined waveform features underscore the excellent sensitivity of the HPCH pressure sensor. Based on these three peaks, the radial augmentation index (*AI*_r_ = *T*/*P*), diastolic augmentation index (*DAI*_r_ = *D*/*P*), and digital volume pulse time (△*T*_DVP_ = *t*_*T−*_*t*_*P*_) were calculated to assess the arterial stiffness of a volunteer, where *t*_*P*_ and *t*_*T*_ represent the time of the first (*P*) and second (*T*) peaks, respectively. The calculated *AI*_r_, *DAI*_r_, and △*T*_DVP_ values under relaxed conditions were 0.545, 0.351, and 0.296, respectively, aligning with expected values for healthy individuals in their mid-twenties [58]. Figures 4d-f and S8 demonstrate the response of the HPCH sensor when applied to various body parts, such as the elbows and knees. As different parts of the body move, the capacitive signals from the HPCH sensor remain strongly synchronized with the corresponding movements, exhibiting both transient and stable responses. This consistency highlights the sensor's performance reliability and reliability in accurately tracking body movements.

Moreover, HPCH-based sensors are not only suitable for health monitoring and medical devices, but also hold great potential in industrial and other applications due to their excellent sensitivity and responsiveness. One promising application is in the fault diagnosis of machinery. For instance, these sensors can monitor the operating state of devices like hair dryers or vacuum pumps by detecting electrical signals generated by shape changes in the sensor when integrated with these machines. Hair dryers are essential household items, widely used in homes and beauty salons, while vacuum pumps are crucial in industrial production for gas extraction and vacuum maintenance, with applications in the chemical, pharmaceutical, and electronics industries. When these devices malfunction, their vibration patterns deviate slightly from normal operation, often leading to abnormal vibrations and noise. The HPCH sensor, when attached to these machines, can detect significant changes in capacitance signals corresponding to these abnormal vibrations, providing an effective means for early fault detection. As illustrated in Fig. 4g, j, we attached the sensor onto the surface of a hairdryer and a vacuum pump to collect dynamic capacitance outputs. To simulate an abnormal working state, we blocked the air inlet, while the open inlet represented normal operation. By analyzing the signal amplitude in the time domain, we could determine whether the machine was functioning properly. Malfunctions are often accompanied by unusual noise, and the unusual noise can be converted by our ultrasensitive sensor into regular capacitance outputs that respond promptly even to multiple simulated malfunctions, as shown in Figs. 4h, k and S9. Additionally, wavelet transformation of the capacitance signal allows for the detection of anomalous states by analyzing not only the frequency information but also the signal intensity (Fig. 4i, l). These experimental results demonstrate that the HPCH pressure sensor holds significant potential for machine fault diagnosis, owing to its ability to detect even the smallest vibration signals.

### Application of HPCH in the Field of Two-Dimensional Pressure Monitoring

**Fig. 5:**
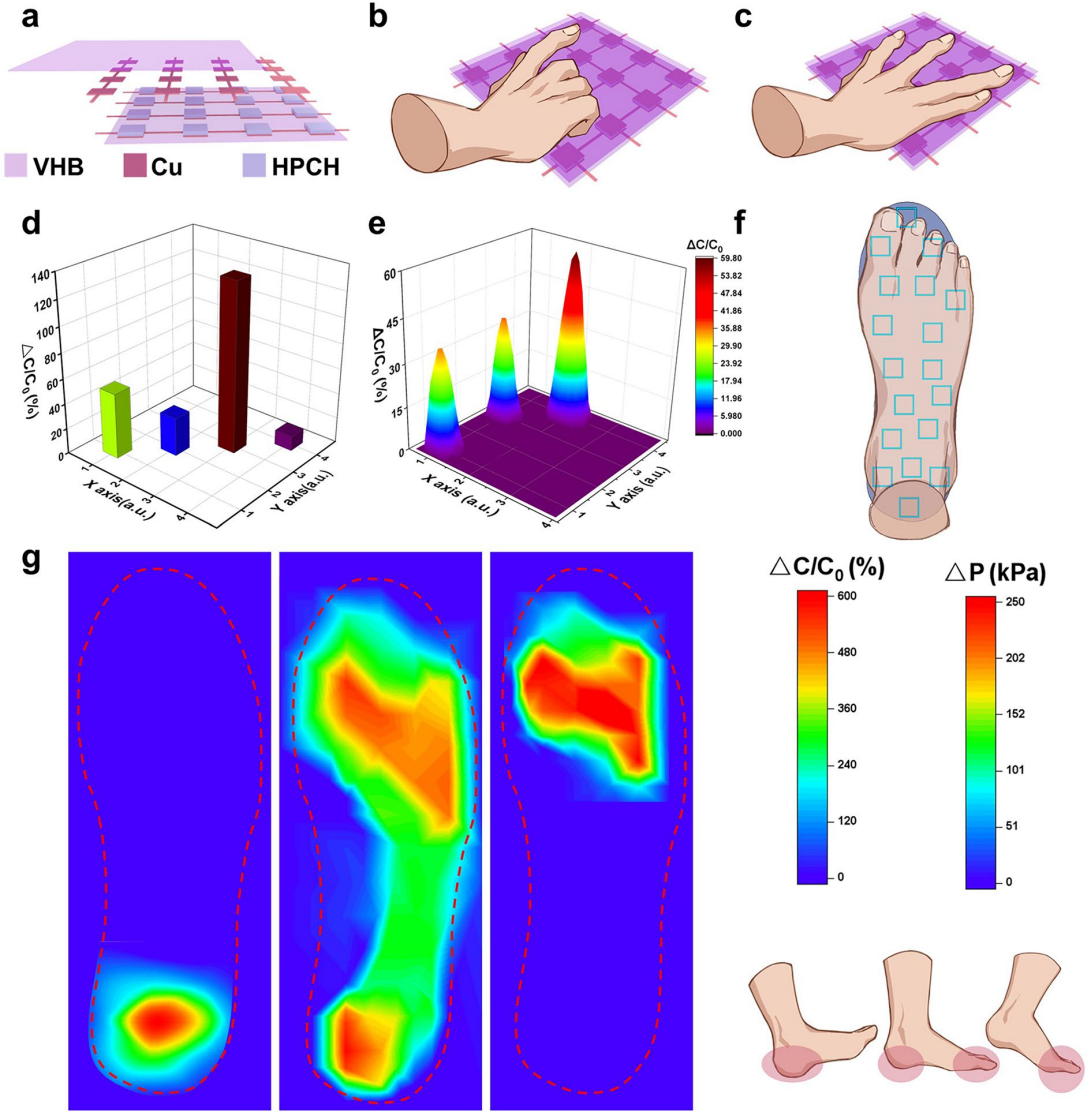
**a** Schematic of the 4 × 4 sensor array for HPCH. **b** Large load size applied to the array with one finger and **d** corresponding signal plots. **c** Different fingers applied to the array and **e** corresponding signal map. **f** Schematic illustration of a plantar pressure monitoring system. **g** Schematic illustrations of three gaits during walking, and the capture of the capacitance signals and pressure maps of the three gaits by using the sensor array

To further explore the wide application of HPCH in the field of pressure sensors, we made a 4 × 4 sensor array based on HPCH to clarify its principle concisely. The simulation diagram of sensor array composition is shown in Fig. 5a with copper sheet as electrodes. The rows and columns were numbered and pressed with different intensities by a single finger at the diagonal position of the sensor array (Fig. 5b). Different pressures were exerted on the hydrogel because of different weights, and then the hydrogel produces different shape variables. Corresponding 3D bar chart of capacitance change is shown Fig. 5d. Next, a volunteer placed three fingers of his right hand on the sensor array at random, applied certain pressure (Fig. 5c). Positions of capacitor change were closely related to the pressed positions. As shown in Fig. 5e, the degree of capacitor change reflected the strength of finger press, where red indicated the largest change, purple indicated the least change. Positions of capacitance change could be matched with the real image precisely, demonstrating HPCH’s ability to accurately sense the magnitude and location of the applied pressure.

The plantar pressure distribution is essential to assess the dynamic loads during human body activities including walking. Therefore, based on the previous simple array, we increased the amount of hydrogel and change the contour shape of the array as a plantar pressure detector, which could provide a variety of important medical data of patients. It was also conducive to disease prevention and surveillance, and the realization of precision medicine. As shown in Fig. 5f, the sensor array was shaped into insoles to monitor the pressure distribution of volunteers when they walked. Supplemented by the walking simulation diagram, Fig. 5g shows the change of pressure distribution in the whole process of walking. The amount of foot pressure is derived from the calibration curve of the pressure versus capacitance change, as shown in Fig. S11. This was also due to the fact that the HPCH sensor encapsulated by the VHB to become more able to withstand pressure, enough to meet the pressure of a person walking.

The pressure distributions of three gaits during walking including heel-strike, mid-stance, and heel-off were recorded. The difference in pressure distribution among the three different gaits can be clearly seen: the pressure concentrates on the center of the heel during heel-strike, and distributes uniformly on both the heel and the forefoot at the mid-stance state with a lower maximal pressure; for the posture of heel-off, the pressure mainly concentrates on the big toe and the forefoot. As such, shoes with such an insole that can dynamically and sensitively detect the pressure distribution of the foot over a wide pressure range would be of great interest in the shoemaking industry and the sports industry. From these above examples, HPCH with high sensitivity has a broad application market despite its simple application principle in the field of pressure sensing.

## Conclusions

This study successfully developed a high-performance cellulose hydrogel, HPCH, based on a biomimetic layered porous structure, demonstrating excellent pressure-sensing capabilities. The distribution of large and small pores within the soft and hard parts effectively balanced the sensitivity and mechanical performance of the material, with the soft part’s high deformability and the hard part’s structural support playing crucial roles during compression. Ion immersion further enhanced the hydrogel’s conductivity and dielectric properties, maintaining high sensitivity across a wide pressure range. The sensor demonstrated a high sensitivity of 1622 kPa⁻^1^, detection range up to 160 kPa, excellent conductivity of 4.01 S m^−1^, rapid response time of 33 ms, and a low detection limit of 1.6 Pa, outperforming most existing cellulose-based sensors. Additionally, the HPCH sensor exhibited outstanding performance in various applications, including health monitoring, machine fault diagnosis, and pressure distribution detection. The findings suggest that the biomimetic layered porous structure offers a promising new pathway for the development of high-performance pressure.

## Supplementary Information

Below is the link to the electronic supplementary material.

Supplementary file1 (DOCX 2070 KB)
