# Supplementary material for: Boosting Sensitivity of Cellulose Pressure Sensor via Hierarchically Porous Structure
Source: Nanomicro Lett. 2025 Mar 31;17:205. doi: 10.1007/s40820-025-01718-z (PMC11958932; doi:10.1007/s40820-025-01718-z)
Supplement: Supplementary file 1 — Supplementary file1 (DOCX 2070 KB) [file 40820_2025_1718_MOESM1_ESM.docx]

Supporting Information for

**Boosting Sensitivity of Cellulose Pressure Sensor via Hierarchically Porous Structure**

Minzhang Chen^1,#^, Xiaoni An^1,#^, Fengyan Zhao^1^, Pan Chen^2,^*, Junfeng Wang^2,^*, Miaoqian Zhang^1^, Ang Lu^1,^ *,

^1^ College of Chemistry and Molecular Sciences, Wuhan University, Wuhan 430072, P. R. China

^2^ School of Material Science & Engineering, Beijing Institute of Technology, Beijing 100081, P. R. China

^#^ Minzhang Chen and Xiaoni An contributed equally to this work.

* Corresponding authors. E-mail: [anglu@whu.edu.cn](mailto:anglu@whu.edu.cn) (Ang Lu); [panchen@bit.edu.cn](mailto:panchen@bit.edu.cn) (Pan Chen); [wjf2015@bit.edu.cn](mailto:wjf2015@bit.edu.cn) (Junfeng Wang)

**Supplementary Figures and Tables**

**Table S1** Deformations corresponding to different parts of HPCH during compression

| Compression deformation of **HPCH** | Compression deformation of **Soft part** | Compression deformation of **Hard part** |
| --- | --- | --- |
| 0 | 0 | 0 |
| 16% | 21% | 4% |
| 39% | 50% | 13% |
| 56% | 62% | 32% |
| 68% | 83% | 55% |

**Table S2** Comparison of HPCH with other materials in sensing properties

| Material composition | Sensitivity (kPa^-1^) | Range (kPa) | Specific conductance (S/m) | Response time (ms) | Detection limit (Pa) |
| --- | --- | --- | --- | --- | --- |
| Cellulose/PVA hydrogel [S1] | 13.91 | 37.3 | 0.46 | - | 14.7 |
| Cellulose ion-conductive hydrogel [S2] | 89.81 | 140 | 3.17 | - | - |
| PAA/Ag^0^ [S3] | 171.4 | - | - | 38 | 0.075 |
| CNT/Ecoflex [S4] | 3.13 | 50 | - | 94 | 0.07 |
| AM / THMA/ VBIMBF4 [S5] | 0.14 | 500 | - | 180 | - |
| Ag/PA@BC aerogel [S6] | 6.92 | - | - | 200 | 28 |
| ACC/PAA/alginate hydrogel [S7] | 1 | - | - | - | - |
| TPU/Ag NW/NaCl [S8] | 1167 | 10.24 | - | - | 1.34 |
| CNTs/GNP Micro@Sponge [S9] | 39.007 | 160 | - | 70 | 0.9 |
| CNT-PDMS [S10] | 372.2 | - | - | 3.33 | - |
| IAH-PET [S11] | 9.32 | - | - | - | 25 |
| TA-SGs [S12] | 9.26 | - | - | 32 | - |
| CF-hydrogel [S13] | 6.01 | - | - | 161 | 1 |
| PVA/MWCNT/CF gel [S14] | 4.2 | 54 | - | 231 | 1.2 |
| This Work | 1622 | 164.707 | 4.01 | 45 | 17 |


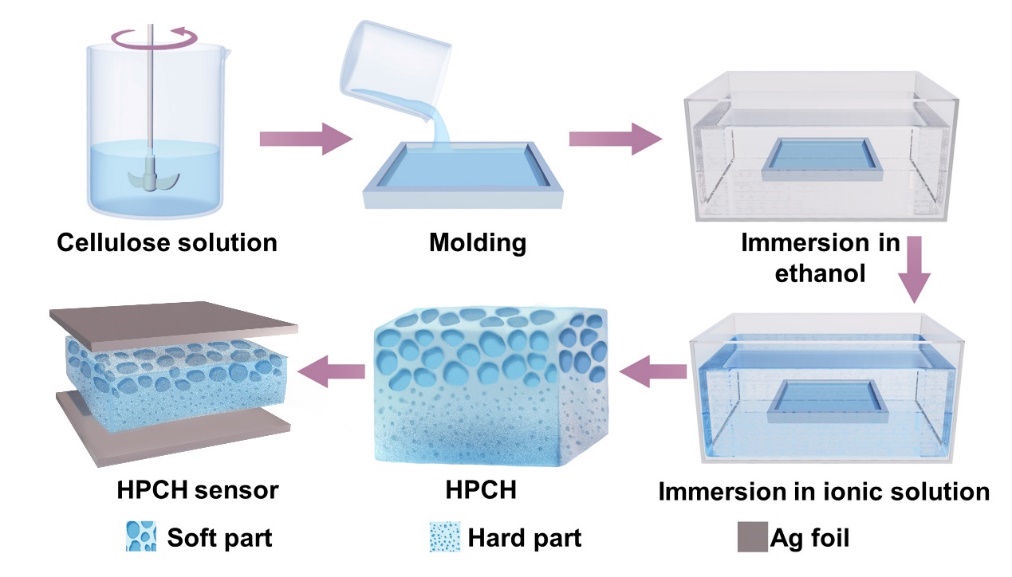


**Fig. S1** Preparation of the HPCH sensor


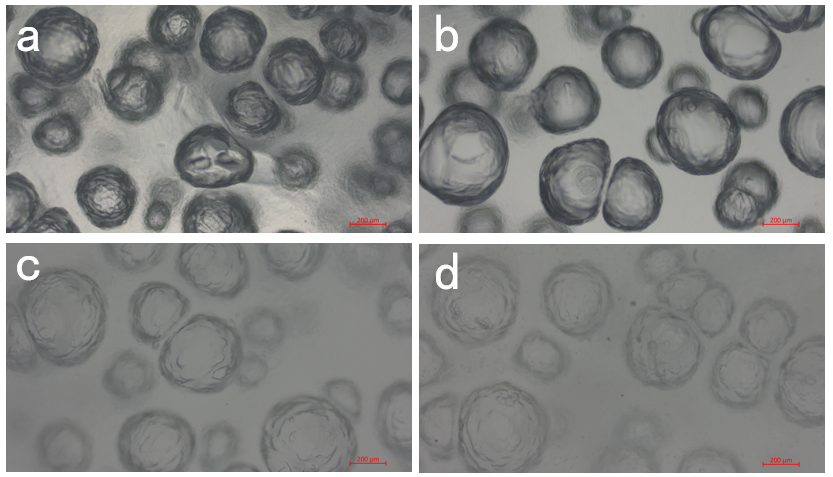


**Fig. S2** Optical micrographs of the upper portion (soft part) of the HPCH immersed in different solutions: (**a**) ethanol; (**b**) KCl, (**c**) LiCl and (**d**) trisodium citrate


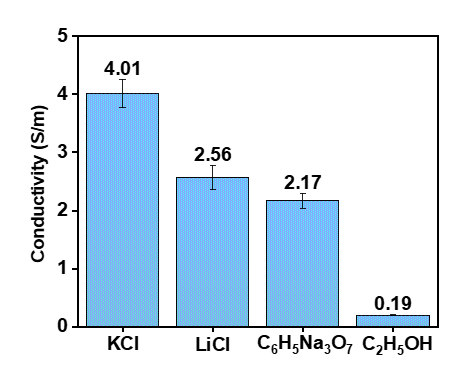


**Fig. S3** Conductivity of HPCHs immersed in different solutions


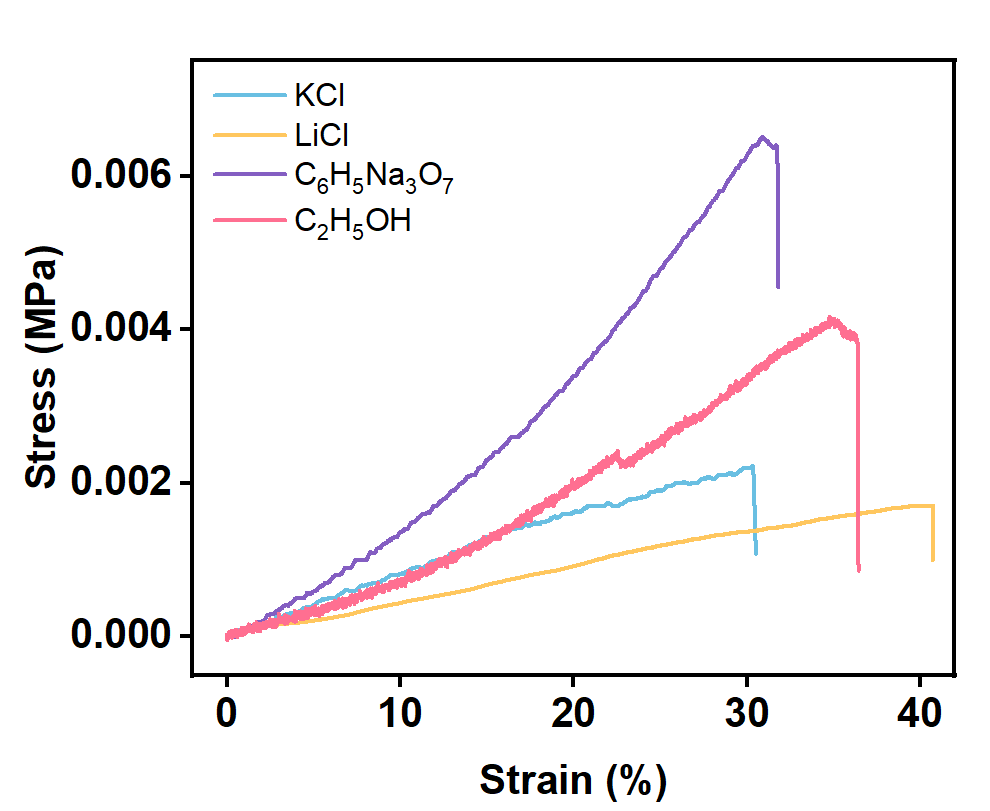


**Fig. S4** Tensile stress-strain curves of HPCHs immersed in different solutions


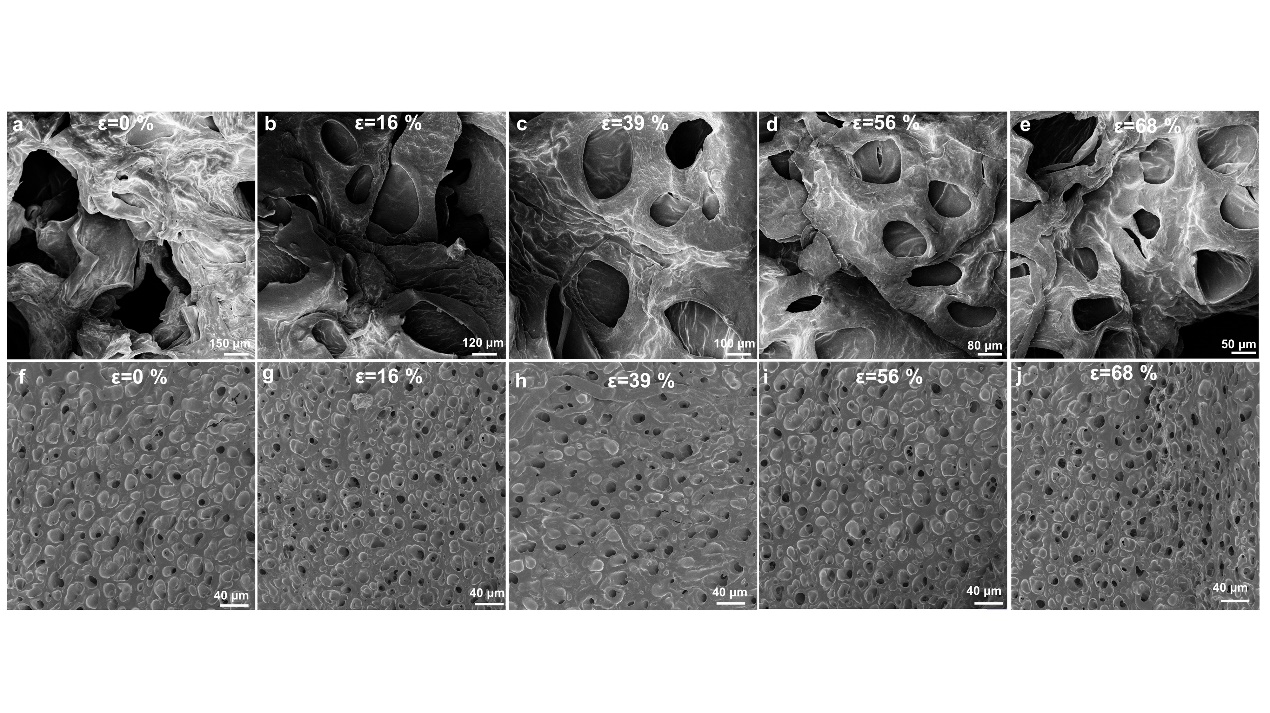


**Fig. S5** SEM images of the soft layer of HPCH during compression with different strains of (**a**) 0%, (**b**) 16%, (**c**) 39%, (**d**) 56%, (**e**) 68%. SEM images of the hard layer of HPCH during compression with different strains of (**f**) 0 %, (**g**) 16 %, (**h**) 39 %, (**i**) 56%, (**j**) 68%


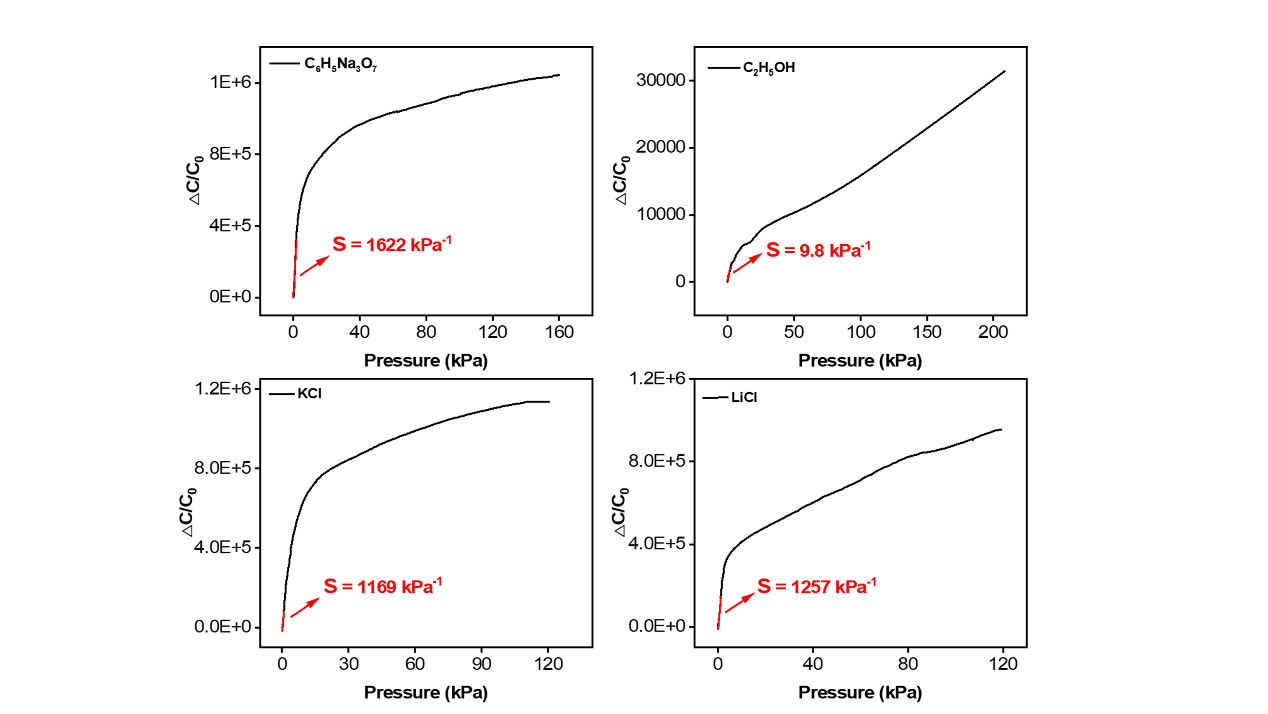


**Fig. S6** Δ*C*/*C*_0_ variations of HPCHs with pressure


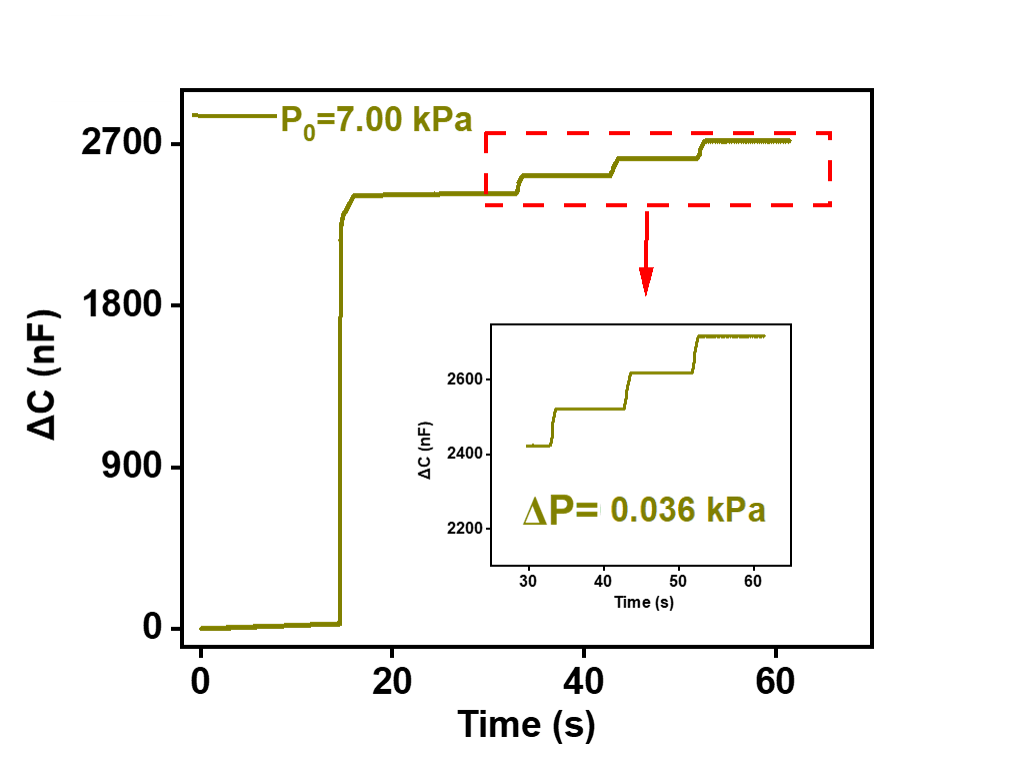


**Fig. S7** Change in capacitance with continued application of a small pressure in a large pressure situation


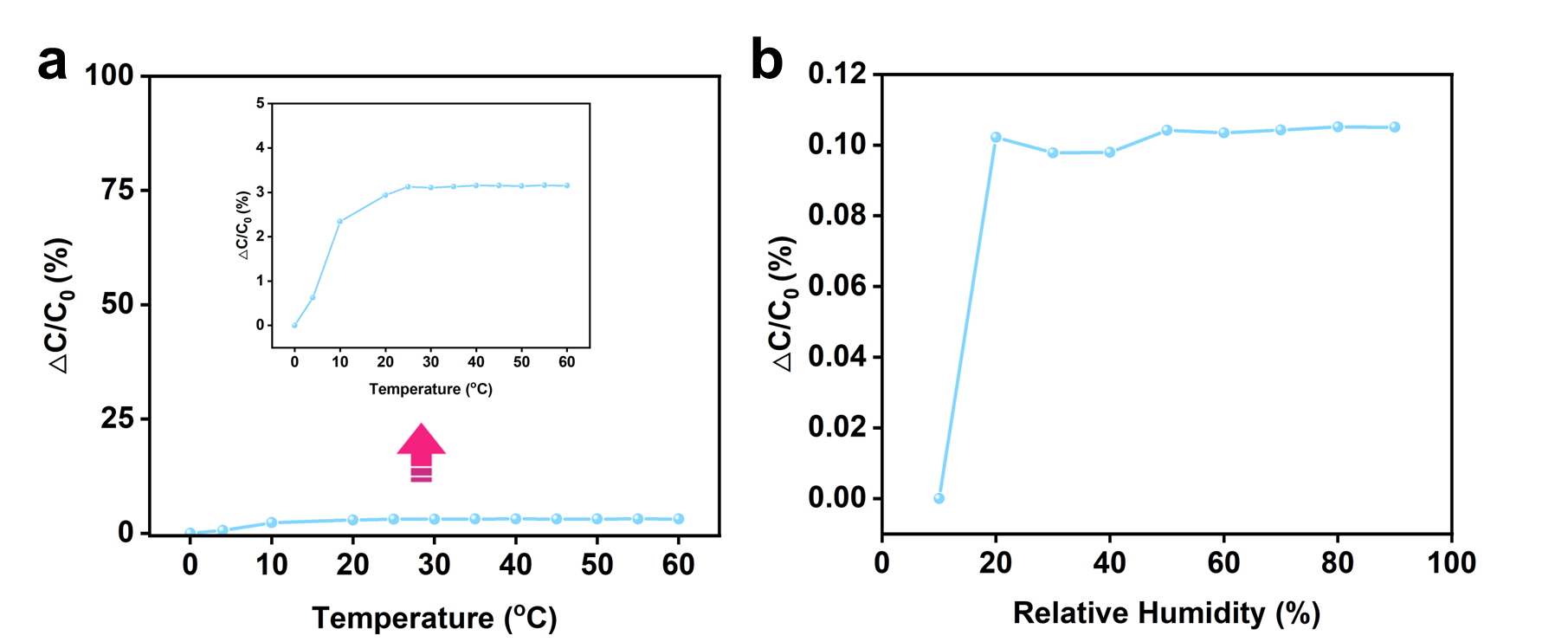


**Fig. S8** (**a**) The capacitance responses of the HPCH sensor at different temperatures (0-60 °C). (**b**) The capacitance response of HPCH sensor in the 10~90 % R.H. range


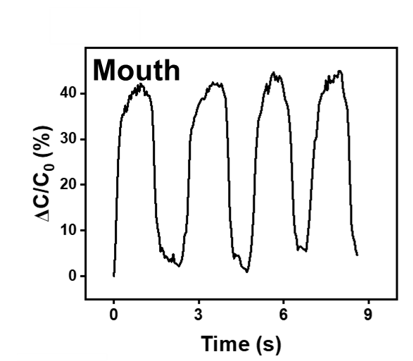


**Fig. S9** The corresponding capacitive signals of mouth


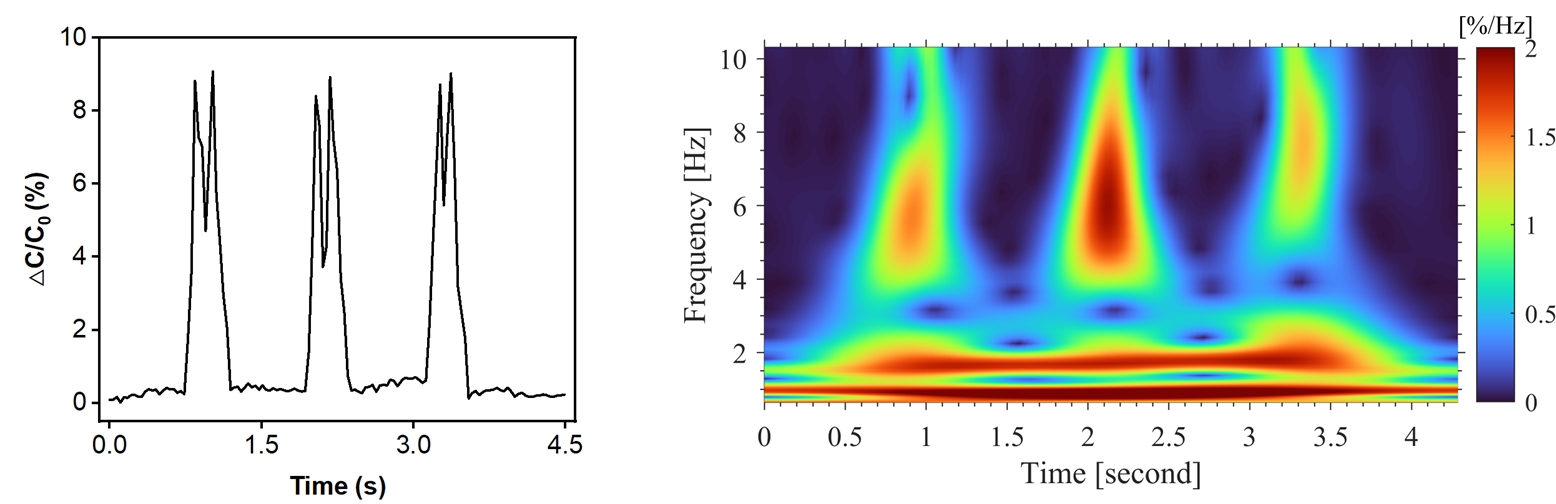


**Fig. S10** Capacitive signals from hairdryer vibrations detected by the sensor, and frequency-time window of capacitive signals from hairdryer vibrations obtained by using wavelet transform


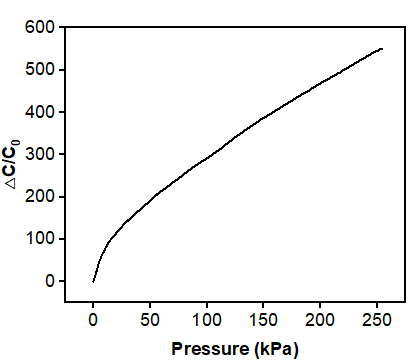


**Fig. S11** Calibration curve for pressure versus capacitance change for HPCH sensor encapsulated by VHB

# Supplementary References

1. Y. Wang, L. Zhang, A. Lu, Highly stretchable, transparent cellulose/pva composite hydrogel for multiple sensing and triboelectric nanogenerators. J. Mater. Chem. A **8**, 13935-13941 (2020). <https://doi.org/10.1039/D0TA02010A>
2. M. Chen, H. Wan, Y. Hu, F. Zhao, X. An et al., Rationally designed cellulose hydrogel for an ultrasensitive pressure sensor. Mater. Horiz. **10**, 4510-4520 (2023). <https://doi.org/10.1039/D3MH01051A>
3. H. Ding, Z. Xin, Y. Yang, Y. Luo, K. Xia et al., Ultrasensitive, low-voltage operational, and asymmetric ionic sensing hydrogel for multipurpose applications. Adv. Funct. Mater. **30**, 1909616 (2020). <https://doi.org/10.1002/adfm.201909616>
4. K.-H. Ha, W. Zhang, H. Jang, S. Kang, L. Wang et al., Highly sensitive capacitive pressure sensors over a wide pressure range enabled by the hybrid responses of a highly porous nanocomposite. Adv. Mater. **33**, 2103320 (2021). <https://doi.org/10.1002/adma.202103320>
5. S. Han, Y. Hu, J. Wei, S. Li, P. Yang et al., A semi-interpenetrating poly(ionic liquid) network-driven low hysteresis and transparent hydrogel as a self-powered multifunctional sensor. Adv. Funct. Mater. **34**, 2401607 (2024). <https://doi.org/10.1002/adfm.202401607>
6. Y. Huang, P. Zhou, X. Zhang, Green synthesis of ag-doped cellulose aerogel for highly sensitive, flame retardant strain sensors. Cellulose **29**, 8719-8731 (2022). <https://doi.org/10.1007/s10570-022-04802-4>
7. Z. Lei, Q. Wang, S. Sun, W. Zhu, P. Wu, A bioinspired mineral hydrogel as a self-healable, mechanically adaptable ionic skin for highly sensitive pressure sensing. Adv. Mater. **29**, 1700321 (2017). <https://doi.org/10.1002/adma.201700321>
8. Y. Liu, J. Tao, Y. Mo, R. Bao, C. Pan, Ultrasensitive touch sensor for simultaneous tactile and slip sensing. Adv. Mater. **36**, 2313857 (2024). <https://doi.org/10.1002/adma.202313857>
9. Y. Ni, L. Liu, J. Huang, S. Li, Z. Chen et al., Rational designed microstructure pressure sensors with highly sensitive and wide detection range performance. J. Mater. Sci. Technol. **130**, 184-192 (2022). <https://doi.org/10.1016/j.jmst.2022.05.021>
10. L. Shi, Z. Li, M. Chen, T. Zhu, L. Wu, Ultrasensitive and ultraprecise pressure sensors for soft systems. Adv. Mater. **35**, 2210091 (2023). <https://doi.org/10.1002/adma.202210091>
11. Y. Tai, Z. Yang, Toward flexible wireless pressure-sensing device via ionic hydrogel microsphere for continuously mapping human-skin signals. Adv. Mater. Interfaces. **4**, 1700496 (2017). <https://doi.org/10.1002/admi.201700496>
12. D. Yang, K. Zhao, R. Yang, S.-W. Zhou, M. Chen et al., A rational design of bio-derived disulfide cans for wearable capacitive pressure sensor. Adv. Mater. **36**, 2403880 (2024). <https://doi.org/10.1002/adma.202403880>
13. H. Yuan, M. Wang, J. Zhang, J. Wang, Y. Le, Hydrogels from chrome shavings for a highly sensitive capacitive pressure sensor. J. Mater. Chem. A **12**, 9797-9805 (2024). <https://doi.org/10.1039/D4TA00702F>
14. H. Yuan, J. Zhang, J. Zhang, M. Wang, J. Wang et al., A “soft and hard” bioinspired hydrogel for enhanced pressure sensing. J. Mater. Chem. C **11**, 10562-10572 (2023). <https://doi.org/10.1039/D3TC01223A>
